# Volumetric MRI Analysis of Brain Structures in Patients with History of First and Repeated Suicide Attempts: A Cross Sectional Study

**DOI:** 10.3390/diagnostics11030488

**Published:** 2021-03-10

**Authors:** Milda Sarkinaite, Rymante Gleizniene, Virginija Adomaitiene, Kristina Dambrauskiene, Nijole Raskauskiene, Vesta Steibliene

**Affiliations:** 1 Department of Radiology, Lithuanian University of Health Sciences, 44307 Kaunas, Lithuania; rymante.gleizniene@lsmuni.lt; 2 Psychiatry Clinic of Lithuanian University of Health Sciences, 44307 Kaunas, Lithuania; virginija.adomaitiene@lsmuni.lt (V.A.); kristina.dambrauskiene@lsmuni.lt (K.D.); vesta.steibliene@lsmuni.lt (V.S.); 3 Laboratory of Behavioural Medicine, Neuroscience Institute, Lithuanian University of Health Sciences, 44307 Kaunas, Lithuania; nijole.raskauskiene@lsmuni.lt

**Keywords:** suicide attempt, FreeSurfer, magnetic resonance imaging, hippocampus, frontal cortex, temporal cortex

## Abstract

Structural brain changes are found in suicide attempters and in patients with mental disorders. It remains unclear whether the suicidal behaviors are related to atrophy of brain regions and how the morphology of specific brain areas is changing with each suicide attempt. The sample consisted of 56 patients hospitalized after first suicide attempt (first SA) (*n* = 29), more than one suicide attempt (SA > 1) (*n* = 27) and 54 healthy controls (HC). Brain volume was measured using FreeSurfer 6.0 automatic segmentation technique. In comparison to HC, patients with first SA had significantly lower cortical thickness of the superior and rostral middle frontal areas, the inferior, middle and superior temporal areas of the left hemisphere and superior frontal area of the right hemisphere. In comparison to HC, patients after SA > 1 had a significantly lower cortical thickness in ten areas of frontal cortex of the left hemisphere and seven areas of the right hemisphere. The comparison of hippocampus volume showed a significantly lower mean volume of left and right parts in patients with SA > 1, but not in patients with first SA. The atrophy of frontal, temporal cortex and hippocampus parts was significantly higher in repeated suicide attempters than in patients with first suicide attempt.

## 1. Introduction

According to the World Health Organization, globally, 780,000 suicide occurrences are recorded each year (10.7 per 100,000 population), and there are from two to three times more suicide attempts [1]. In Lithuania, suicide is one of the major public health issues. It should be noted that Lithuania has been one of the countries with the highest suicide rate for more than 20 years [2]. With the rate of 31.9/10,000 Lithuania is in the first place in the European Union in terms of the number of suicides. It is three times higher compared to the rest of the world and twice as high as recorded in the EU (15.4 per 100,000 population) [1]. Therefore, in Lithuania, as well as in the whole world, great importance is attached to identifying both psychosocial and neurobiological factors of suicidal behaviors for the purpose of effective suicide prevention.

A number of neurobiological models have been proposed to explain the suicidal behaviors [3]. The identification of these models could be the effective step in suicide prevention. Nowadays structural neuroimaging is one of the best modern methods for examinations of suicidal behaviors in the living brain [4]. There are more than 30 studies known which indicate that structural brain changes are found in suicide attempters, they also reveal how variation in brain volume is associated with pathogenesis of suicide [4]. However, data are quite contradictory; brain changes in most studies are more related to mental disorders than suicidal behaviors [4]. Atrophy of dorsolateral (DL) prefrontal cortex (PFC), ventrolateral (VL) areas of PFC and anterior cingulate cortex (ACC) is found in suicide attempters, the patients with depression or schizophrenia [5,6]. Other studies on suicidality detected grey matter atrophy in the left superior temporal gyrus, left orbitofrontal cortex (OFC), superior left parietal lobe, thalamus, insula of the right side, and some frontal regions [6,7,8,9]. In contrast, increased volumes of the right amygdala and the reciprocal inferior frontal white matter are also discovered in suicide attempters [6,7,8,9]. Research on suicidal behaviors in patients with unipolar disorder found support to volume reduction in right and left OFC, hippocampal volume in comparison to healthy controls [10].

To the best of our knowledge, there has been no investigation into brain volume changes after first and repeated SA. Thus, it is remains unclear, how the morphology of specific brain areas changes with each subsequent suicide attempt. In this cross-sectional study we examined structural differences in temporal cortex (TC), frontal cortex and hippocampal regions between patients with history of first and repeated SA in comparison to healthy controls by using brain magnetic resonance imaging (MRI) and automatic segmentation technique.

## 2. Materials and Methods

### 2.1. Study Population and Settings

Adults, non-psychotic patients, men and women, without cognitive impairment, any other organic brain disorder or severe unstable medical condition, admitted to Psychiatry department after SA during a six-month period, eligible for MRI scan were invited to participate in this cross-sectional study. All study patients completed a questionnaire that included assessment of sociodemographic characteristics, history of psychiatric treatment and hospitalization and history of suicide attempts.

Psychiatric diagnoses and suicide attempts were evaluated by a psychiatrist and defined under the Diagnostic and Statistical Manual of Mental Disorders 5th edition (DSM-5) [11]. Depressive symptoms of the study patients were evaluated by psychiatrist using the structured interview guide for the Montgomery–Asberg Depression Rating Scale (MADRS-SIGMA) (Copyright ©2006 v.1, Janet B.W. Williams and Kenneth A. Kobak. All rights reserved.) [12]. This scale consisted of 10 questions and ratings were based on the patient’s condition in the past week. Answers were rated in 7-point scale (0—no symptoms, 6—very extreme symptom severity); the sum of all ratings provided the severity of depressive symptoms. SA has been defined as a nonfatal self-directed, potentially injurious behavior with any intent to die as a result of the behavior [13]. The control group consisted of adult subjects (men and women), who consecutively came for preventive check-ups to a family physician at the Clinic of Family Medicine. The exclusion criteria for control group were the anamnesis of mental disorder, alcohol or drug abuse or dependence, anamnesis of SA, cognitive impairment, severe unstable medical conditions, any organic brain disorders. The study protocol and informed consent procedures were approved by the Bioethics Committee at the Lithuanian University of Health Sciences, Kaunas, Lithuania (2017-06-23, No. BEC-LSMUCR-67). The investigation was carried out in accordance with the Declaration of Helsinki. Each study participant gave written informed consent prior to all study procedures.

Of all invited as study patients, 59 subjects (response rate 92%) agreed to participate in the study. One patient was excluded due to specific MRI changes of carbon monoxide poisoning and 2 patients were excluded due to anamnesis of chronic alcohol consumption that could had caused brain changes. The healthy control (HC) group included 54 volunteers (response rate 68%). Hence, the final study sample consisted of 56 study patients (mean age 40.8 (16.8) years, 35.2% of them were men) after SA and 54 HC (mean age 41.0 (16.3) years, 39.3 % were men). There were no significant differences between patients and controls with regard to age and gender (Table 1). Of the 56 study patients included into final analysis, 21 (37.5%) were diagnosed with major depressive disorder (MDD) (MADRS score 28.14 (7.9)); other – 26 (46.43%) with adjustment disorder, 3 (5.36%) with personality disorders and 6 (10.71%) with anxiety disorders (MADRS score in this group was 9.34 (1.69)).

### 2.2. MRI Processing and Analysis

Patients after SA and HC went through the same MRI protocol, which consisted of T2-weighted (T2W)/FLAIR axial (TR 9000ms, TE 89ms) and T2W/fl2d/hemo axial (TR 800ms, TE26ms) sequences in order to rule out the pathologies as lesions and hemorrhage. Image data were collected using 1.5 Tesla (T) Siemens Avanto (Siemens, Munich, Germany) scanner with a standard quadrature head volume coil. Head movement was prevented by pads inside the coil. Whole-brain T1-weighted (T1W) mpr/p2/iso axial (TR 1900ms, TE 3.35ms, 1 mm slice thickness) sequence was used for volumetric measurement. During brain MRI, the patients had no suicidal ideation.

All MRI data were subject to strict quality control procedures. First, the radiologists checked T1W images for artefacts. If the latter met the requirements, they were converted to mgz files. Brain cortical thickness and grey- and white-matter volumes were measured using FreeSurfer 6.0 image analysis suite (http://surfer.nmr.mgh.harvard.edu/; accessed on 22 September 2018). This automatic reconstruction technique outweighs standard brain volume measurement due to the fact that it investigates across the whole brain and can locate changes which may be very small or do not match usual anatomical subdivisions. The FreeSurfer software constructs a three-dimensional representation of the cortical surface and volume quantifications applying image intensities and continuity data from the entire MRI volume to create reproductions of the border between gray and white matter and pial surface [4]. FreeSurfer’s Desikan–Killiany atlas was used to define regions of interest for the brain cortex: the frontal cortex contained 11 regions, temporal cortex—9 regions [14,15]. Freesurfer‘s automated segmentation of hippocampal substructures divided the hippocampus into 13 subfields [16].

### 2.3. Statistical Analysis

The Shapiro–Wilk test was employed to verify whether the data meet the conditions of the normal distribution. The comparison between all patients after SA and comparison control subjects was performed using two-sample t-tests. Observed differences were considered statistically significant when the calculated significance level (*p*-value) was lower than the selected significance level (α = 0.05). Values for the average cortical thickness were analyzed using a general linear model controlling for the effect of age. ANOVA was used to test the equality of three group means, statistically significant results indicate that not all of the group means are equal. Post hoc tests were used to explore differences between multiple group means. Frontal and TC thickness as well as hippocampus volume was compared among the three groups (control, first SA, SA>1) using analysis of covariance (ANCOVA), with post hoc pairwise comparisons for age as covariate of interest. The *p*-value was used in the ANOVA output to determine whether the differences between some of the means are statistically significant. F values are based on the pairwise comparisons among the means. *p*-values were adjusted for multiple comparisons using Bonferroni correction for multiple comparisons. An additional entire cortex analysis was performed in order to investigate a potential gender by group interaction in cortical thickness. Covarying for gender in the full-group analyses did affect the results. Thus, the final analyses were performed without covarying for gender. Alpha levels for statistical significance testing were set to 0.05. IBM SPSS Statistics 23 and Microsoft Excel 2010 were used for statistical analysis.

## 3. Results

All study patients, hospitalized after SA (*n* = 56) were divided into the following two groups: suicide attempters with a history of at least one suicide attempt (first SA group, *n* = 29), and suicide attempters who reported more than one suicide attempt during the lifetime (SA > 1 group, *n* = 27). As reported in Table 1, the groups did not differ significantly in age, gender, main sociodemographic characteristics, psychiatric diagnoses, severity of depressive symptoms or method of suicide attempt, but they did vary in terms of marital status, with a greater proportion of single participants in the first SA group. SA>1 group had significantly longer duration of mental disorder (*p* < 0.001) in comparison to the first SA group. No differences were found between these groups regarding the psychiatric treatment 1 month before SA and period of time between last hospitalization and current SA. All patients with SA > 1 reported psychiatric consultations during lifetime (more frequently than patients after the first SA, *p* = 0.005).

The volumetric analysis of all brain structures was done. Overall, significant differences between all SA patients and healthy controls groups were found in frontal and TC thickness and in volume of the left and right parts of hippocampus. Decreased cortical thicknesses were observed in both hemispheres. Analysis of other brain regions did not reveal significant results.

The analysis of frontal cortex thickness is shown in Table 2. The comparison between the patients after first SA and HC revealed the significant lower cortical thickness of the superior frontal (*p* = 0.041) and rostral middle frontal (*p* = 0.016) areas of the left hemisphere and superior frontal area (*p* = 0.021) of the right hemisphere, with relative 3.5, 3.58 and 4.19% difference of mean cortical thickness in these cortex areas, respectively. Moreover, the comparison between the SA > 1 group and controls revealed the significant lower frontal cortical thickness in ten areas of the left hemisphere and seven areas of the right hemisphere, with relatively higher difference (4.02 to 8.33%) of the mean cortical thickness between these two groups. Differences in four of frontal cortex regions were highly significant (*p* < 0.0001) in patients with SA>1 as compared with the control group.

Table 3 presented the comparison of thickness of temporal and frontal cortex among study groups. Patients after first SA, in comparison to HC, had significantly lower cortical thickness of the inferior (*p* = 0.002), middle (*p* = 0.013) and superior (*p* = 0.006) temporal areas of the left hemisphere, with relative 4.09, 4.02 and 4.49% difference of mean cortical thickness in these cortex areas, respectively. In patients with SA > 1, in comparison to controls, the significant lower TC thickness was observed in the six areas of both hemispheres, with a difference (3.90 to 6.04%) of the mean cortical thickness between these two groups. Differences in the inferior and middle temporal areas of the left hemisphere were observed as highly significant (*p* < 0.001).

Significant differences in volume of left and right parts of hippocampus between study controls and all study patients are demonstrated in Table 4.

Analysis of patients with history of first SA did not reveal significant differences in volume of hippocampus parts, in comparison to HC (Table 5). However, the comparison of controls to patients with SA > 1 reported significant differences in mean volume of left and right parts of hippocampus volume, with 7.86 to 9.89% relative differences of the mean hippocampus volume between two groups.

Post-hoc analyses revealed no significant within group differences in brain thickness and hippocampus parts volume in the groups of patients after SA, when comparing first SA group (*n* = 29) to SA > 1 group (*n* = 27).

The comparison of frontal and temporal cortex thickness and the volumes of hippocampus parts of all study patients with history of SA according to psychiatric diagnoses (major depressive disorder vs. other diagnosis), method of suicidal attempt (intoxication with drugs vs. physical suicidal act) and acute alcohol intoxication before SA (yes vs. no) did not reveal significant differences.

## 4. Discussion

To our knowledge, it is the first study, which compared volumetric brain parameters in acute suicidal patients after first and repeated suicidal attempts to healthy subjects without a history of suicidal attempt and mental disorder. The main finding of this research was that already after the first suicide attempt in comparison to healthy controls lower cortical thickness was observed in temporal and frontal areas; smaller size of some parts of hippocampus structure was found in suicidal patients.

According to the data of literature, about 90% of suicide attempters usually had some kind of untreated psychological disorder, 50–70% of the time that disorder was depression [17,18]. In our study as many as a third of the patients had MDD, but more than half had records of psychiatric hospitalization and previous psychiatric treatment. Women, as a group, demonstrated more attempts, which can be explained by a weaker intention to die, but a stronger desire to communicate distress [19]. However, the majority of patients did not had records of suicide in family which was linked to self-harm in other studies by genetic transfer [20,21]. On the other hand, more than half of subjects were diagnosed with adjustment disorder and experienced a recent stressful life event, which is a risk factor for suicidal behaviors, as it frequently precedes a suicide attempt [22]. Our study showed that married people were more likely to repeat a suicide attempt. It suggests that if a person feels emotionally unsupported being married or living in a couple, relationship status could not serve as a protective factor for suicidal behaviors. This supports other authors data that multiple suicide attempters tend to be single [23] and have middle economic status [24]. Other studies state that low income contributes to more SA, this question is debatable and relies more on country economic status [25,26]. It was found that more SA lived in urbanized area, as in study of Qin et all (2003), but contradicts to Turecki et all (2016), which stated that rural areas are associated with three times the risk of suicide [24,27].

Volumetric brain analysis in our study revealed that frontal cortex thickness was reduced in rostral middle frontal region on the left hemisphere and superior frontal regions in both hemispheres. Moreover, these differences in cortical thickness were significantly higher in the assessment of patients after repeated suicide attempts: the significant difference in cortical thinning of almost all frontal regions was found.

Many studies investigated frontal cortex role in suicidality. It is known that DL, VL areas of PFC and ACC regulate behaviour and emotions [28]. The aforementioned changes lead to impaired behavioural control and impulsive behaviour. It is thought that in an emotionally unstable person the frontal cortex limbic system inhibition is not sufficient enough, which causes impulsive, irrational decision making and emotional liability in individuals [29,30,31]. In the findings of other researchers, cortex thinning in PFC and OFC areas was linked with suicidality [32,33]. Atrophy in prefrontal cortex ventrolateral, dorsolateral and anterior cingulate parts was also discovered in MDD, bipolar depression and schizophrenia patients who attempted suicide [5,6,34]. Study of Wang et al. found that MDD patients with a history of SA had a reduced volume in the right and left amygdala and ventral, medial, dorsal PFC. These demonstrate the role of the amygdala and PFC in the pathogenesis of suicidal behavior and imply the amygdala-PFC circuit as a probable target for detection and prevention of suicidal behaviors [35].

Our study has also revealed significant differences in superior frontal gyrus, rostral middle frontal gyrus, pars triangularis and precentral gyrus and other areas of frontal cortex. It has been investigated that pars triangularis is associated with cognitive control of memory and may contribute to suicidal behaviors through a connection with hippocampus [36]. The prefrontal cortex is also linked to suicidality because of its role in executive functions [37]. Other frontal cortex regions involved in suicide have not yet been investigated.

Furthermore, our investigation of temporal lobe structures revealed reduced cortical thickness in inferior, middle and superior regions in patients with first suicidal attempt and nearly all TC regions—in patients with repeated suicidal attempts. It is known about the association of TC with limbic structures and the PFC: these interfaces are important in recognizing and controlling mood and emotion [38]. Superior temporal gyrus is related to emotional intelligence, the ability of following own and others’ emotions to make decisions and perform actions [6]. Superior temporal gyrus and sulcus are the components of the face recognition system [39]. This gyrus becomes active upon seeing scared faces [40]. Superior temporal sulcus, amygdala and insula analyse movements of other objects, providing information about their intentions [41]. In addition, these structures regulate a sudden, reflexive response to negative visual stimuli [42]. In other studies, the role of TC in suicidality has been mainly linked to degradation in medial and superior cortices [6,33,40,43,44]. This could be due to the fact that temporal lobe contributes to emotion responding [45]. Our research has demonstrated significant volumetric results in the previously mentioned middle, superior TC and also in inferior, left fusiform and left temporal pole. Similar findings were reported in other studies that investigated fusiform relation to borderline personality disorder, while smaller temporal pole is associated with psychotic disorders and suicide attempts [40,46].

One of the aims of our study was to examine hippocampus structure differences in patients who attempted suicide. This structure is known to be associated with the pathophysiology of mental disorders, while the main function of the hippocampus is memory processing. Changes in this cognitive function were associated with suicidal behaviors and lower hippocampal volume in individuals who attempted suicide [47]. As expected, our study demonstrated that suicide attempters showed significant volume differences in some left and right parts of hippocampus structures. However, some hippocampus regions were found as reduced only in patients after repeated SA, without significant differences in patients after first suicidal attempt in comparison to controls. Our findings supported the Colle et al. study in which depressed suicide patients had smaller right and total hippocampus volume than non-suicidal attempters. Hippocampal volumes were negatively associated with impulsivity in individuals; high lethality attempts were associated with smaller volumes of the hippocampus and parahippocampal gyrus in adult suicidal attempters [40,48]. Other study of Gosnell and colleagues found smaller hippocampus in depressed suicidal patients in comparison to healthy controls. However, they did not find differences in hippocampus of suicidal and not suicidal depressive patients. Researchers hypothesized that hippocampus volume differences could be due to a characteristic other than suicidality [32]. Sapolsky’s theory is based on stress model—hippocampus could be damaged by stress-induced release of steroidal and inflammatory substances [49]. It has been also found that hippocampus modulates the activity of the hypothalamic–pituitary–adrenal axis which is impaired in people who have attempted suicide, not to mention the prefrontal cortex which performed executing functions and is regulated by the hippocampus [50,51]. As mentioned before, the majority of suicide patients in this study experienced a stressful life event one month before SA or were diagnosed with adjustment disorder. We believe that stress might be a risk factor for the atrophy of hippocampus and it could lead to suicidal behaviors. This lends support to previous findings in the literature suggesting that acute stressing event and high levels of cortisol could associate with decreased volume of the hippocampus [52].

A growing body of literature has discovered that mental disorders, especially MDD and schizophrenia, are related to suicidal behaviors [18,53]; affective disorders comprise more than half of all suicide deaths [54]. In our study, only one-third of patients were diagnosed with MDD, other patients had adjustment disorder, anxiety disorders and personality disorders. However, our study results revealed that reduced brain volume in suicidal patients was completely unrelated to neither age, gender, method of suicide attempt or diagnosis of MDD. Most of the volumetric studies evaluating suicidal patients provided observations when comparing suicidal vs. non-suicidal patients with an affective or psychotic disorder. Hippocampus volume reduction was one of the most frequently reported finding, associated with MDD, however changes in size of hippocampus remain unclear in suicide attempters without known mental or neurological disorder [55,56,57]. Smaller hippocampus in patients with MDD was related to the levels of serum brain-derived neurotrophic factor (BDNF) and it may be a significant underlying factor in hippocampal volume differences between patients with MDD and healthy subjects [58]. On the contrary, other authors emphasized that the diagnosis of MDD was not significantly related to hippocampal volume. Our study did not reveal any significant hippocampal volume differences between suicide attempters with and without MDD and our results let us to emphasize that suicidal behaviors but not on MDD may influence the changes in size of hippocampus [59].

Frodl et al. stated that reduction of hippocampus in patients with MDD cannot be confirmed during the three-year follow-up period, but he underlined that atrophy of hippocampus may contribute to the development of MDD and poor clinical outcome in patients [59]. Hippocampus volume changes in suicidal behaviours remained contradictory [60]. Hence, at this stage of understanding, we hypothesized that the model of suicidal behaviours is related to the reduction of brain volume in specific brain regions—frontal, temporal cortex and hippocampal structures. Moreover, these changes become express with repeated suicidal attempts.

This study has some limitations. The first is a cross-sectional study design. This bears importance because differentiating the cause and effect becomes impossible—we are unable to tell whether reduced brain volume contributes to suicidal behaviours or the opposite. The second is that we were unable to compare brain volumetric data in patients with mental disorders without a history of prior suicide attempt. The large sample was useful for exploring hippocampus changes after first suicidal attempt. The prospective analysis of structural brain changes after each repeated SA could provide more clear information about neuroimaging markers of the suicidal brain.

## 5. Conclusions

We concluded that acute hospitalized suicide attempters had lower cortical thickness in frontal and temporal brain regions of and smaller parts of hippocampus than healthy subjects, without history of suicide attempt and mental disorder. These differences were found independently to patients’ age, gender or diagnosis of depressive disorder. The atrophy of frontal, temporal cortex and hippocampus parts was significantly higher in repeated suicide attempters than in first suicide attempters. These findings suggest that repeated suicidal behaviors are associated with intensifying changes in specific brain structures.

## Figures and Tables

**Table 1:** Demographic characteristics of all study subjects.

Characteristics	HC (*n* = 54)	All Patients after SA (*n* = 56)	p^a^	First SA Group (*n* = 29)	SA > 1 Group (*n* = 27)	F/*p* Chi-Square
Age, mean (SD), years range	41.0 (16.3) 18–77	40.8 (16.8) 18–74	0.955	41.1 (18.1)	40.6 (15.6)	0.993 ^a^
Gender, M/F, *n* (%)	19/35 (35.2/64.8)	22/34 (39.3/60.7)	0.656	14/15 (48.3/51.7)	8/19 (29.6/70.4)	0.320 ^a^
Residential area, rural/urban, *n* (%)	-	11/45 (19.6/80.4)		3/26 (10.3/89.7)	8/19 (29.6/70.4)	0.070
Education, university/college/secondary school, *n* (%)	-	13/29/14 (23.2/51.8/25)		7/16/6 (24.1/55.2/20.7)	6/13/8 (22.2/48.1/29.6)	0.740
Marital status, married/single, *n* (%)	-	27/29 (48.2/51.8)		10/19 (34.5/65.5)	17/10 (63.0/37.0)	0.033
Having children, Yes/no, *n* (%)	-	31/25 (55.4/44.6)		14/15 (48.3/51.7)	17/10 (63.0/37.0)	0.269
Economic status, low/ middle/ high, *n* (%)	-	15/32/9 (26.8/57.1/16.1)		7/17/5 (24.1/58.6/17.2)	8/15/4 (29.6/55.6/14.8)	0.891
Family history of suicide, Yes/no, *n* (%)	-	8/48 (14.3/85.7)		2/27 (6.9/93.1)	6/21 (22.2/77.8)	0.104
Stressful life event one month before last SA, Yes/no, *n* (%)	-	34/22 (60.7/39.3)		21/8 (72.4/27.6/)	13/14 (48.1/51.9/)	0.063
Duration of mental disorder, mean (SD), years	-	3.45 (2.41)		2.38 (2.06)	4.59 (2.26)	<0.001
Previous psychiatric hospitalization, Yes/no, *n* (%)	-	37/19 (66.1/33.9)		18/11 (62.1/37.9)	19/8 (70.4/29.6)	0.512
Period between last hospitalization and current SA, mean (SD), years	-	1.0 (0.83)		0.93 (0.84)	1.07 (0.83)	0.525
Psychiatric consultation during lifetime, Yes/no, *n* (%)	-	48/8 (85.7/14.3)		21/8 (72.4/27.6)	27/0 (100/0)	0.005
Psychiatric treatment 1 month before last SA, Yes/no (%)	-	37/19 (66.1/33.9)		16/13 (55.2/44.8)	21/6 (77.8/22.2)	0.074
Current psychiatric diagnosis, MDD/ other^b^, *n* (%)	-	21/35 (37.5/62.5)		10/19 (34.5/65.5)	11/16 (40.7/59.3)	0.418
Age at first SA, years	-	38.2 (17.2)		41.1 (18.1)	35.1 (15.9)	0.201
History of SA, First time/repeated, *n* (%)	-	29/27 (51.8/48.2)		–	–	
Method of SA, drugs/physical, *n* (%)	-	31/25 (55.4/44.6)		16/13 (55.2/44.8)	15/12 (55.6/44.4)	0.595
Alcohol intoxication during SA, Yes/no, *n* (%)	-	22/34 (39.3/60.7)		12/17 (41.4/58.6)	10/17 (37/63)	0.477
Smoking, Yes/no, *n* (%)	-	24/32 (42.9/57.1)		10/19 (34.5/65.5)	14/13 (51.9/48.1)	0.189
MADRS score, mean (SD), range	-	16.39 (10.43) 7–47		16.07 (10.02) 7–43	16.74 (11.04) 7–47	0.489

HC, healthy controls; SA, suicide attempt; SD, standard deviation; M, male; F, female; MDD, major depressive disorder; MADRS, Montgomery–Asberg depression rating scale; p^a^ value between three groups: HC, first SA, SA > 1. ^b^ other diagnoses include: anxiety disorder, borderline personality disorder, adjustment disorder. Significant *p*-value (*p* < 0.05).

**Table 2:** The significant differences in means of frontal cortex thickness between the patients after first suicide attempt, repeated suicide attempts and study controls (first SA, SA > 1, HC).

Hemisphere	Frontal Cortex Part	Mean Cortical Thickness (95% CI) in mm							ANCOVA Age Adjusted	
		HC (*n* = 54)	First SA (*n* = 29)	Post-Hoc	% Diff.	SA > 1 (*n* = 27)	Post-Hoc	% Diff.		
		1	2	1 vs. 2	1–2	3	1 vs 3	1–3	F_2.106_	*p*
Left	Lateral orbitofrontal	2.79 (2.75–2.83)	2.72 (2.66–2.77)	0.140	2.50	2.66 (2.60–2.72)	0.002	4.65	6.71	0.002
	Medial orbitofrontal	2.55 (2.50–2.58)	2.54 (2.49–2.60)	1.000	0.39	2.42 (2.36–2.48)	0.003	5.09	6.74	0.002
	Pars opercularis	2.66 (2.61–2.72)	2.58 (2.51–2.65)	0.149	3.00	2.48 (2.41–2.55)	<0.001	6.77	11.29	<0.001
	Pars orbitalis	2.88 (2.82–2.94)	2.78 (2.70–2.86)	0.142	3.47	2.69 (2.61–2.77)	0.001	6.59	7.32	0.001
	Pars triangularis	2.56 (2.51–2.61)	2.49 (2.43–2.56)	0.329	2.73	2.41 (2.34–2.48)	0.002	5.85	6.40	0.002
	Precentral	2.52 (2.46–2.56)	2.42 (2.35–2.48)	0.093	3.96	2.31 (2.24–2.38)	<0.001	8.33	10.34	<0.001
	Rostral middle frontal	2.51 (2.47–2.54)	2.42 (2.37–2.47)	0.016	3.58	2.37 (2.32–2.42)	<0.001	5.58	11.48	<0.001
	Superior frontal	2.85 (2.81–2.90)	2.75 (2.68–2.81)	0.041	3.50	2.66 (2.59–2.73)	<0.001	6.67	11.11	<0.001
	Frontal pole	2.84 (2.76–2.91)	2.76 (2.65–2.87)	0.765	2.81	2.66 (2.55–2.77)	0.032	6.33	3.43	0.036
	Caudal middle frontal	2.64 (2.59–2.69)	2.54 (2.48–2.61)	0.058	3.78	2.50 (2.43–2.69)	0.002	5.3	6.78	0.002
Right	Lateral orbitofrontal	2.73 (2.68–2.77)	2.69 (2.63–2.75)	0.944	1.46	2.62 (2.56–2.68)	0.013	4.02	4.28	0.016
	Pars opercularis	2.63 (2.57–2.68)	2.57 (2.49–2.65)	0.824	2.28	2.48 (2.40–2.56)	0.011	5.70	4.39	0.015
	Pars orbitalis	2.86 (2.79–2.91)	2.81 (2.72–2.89)	1.000	1.74	2.71 (2.62–2.80)	0.026	5.24	3.58	0.031
	Pars triangularis	2.55 (2.49–2.60)	2.51 (2.44–2.58)	1.000	1.56	2.42 (2.35–2.49)	0.016	5.09	4.07	0.020
	Precentral	2.46 (2.41–2.51)	2.39 (2.32–2.46)	0.290	2.84	2.31 (2.24–2.38)	0.002	6.09	6.42	0.002
	Rostral middle frontal	2.46 (2.41–2.50)	2.42 (2.36–2.49)	1.000	1.62	2.35 (2.92–2.42)	0.040	4.47	3.19	0.045
	Superior Frontal	2.86 (2.81–2.91)	2.74 (2.68–2.81)	0.021	4.19	2.68 (2.62–2.75)	<0.001	6.29	9.58	<0.001
	Caudal middle frontal	2.59 (2.54–2.64)	2.51 (2.44–2.58)	0.267	3.08	2.48 (2.40–2.55)	0.054	4.24	3.33	0.039

HC, healthy controls; SA, suicide attempt. F, *p* values are based on the pairwise comparisons among the means (three groups: HC, first SA, SA > 1). % diff—relative difference describes the difference of the mean cortical thickness between two groups: HC and patients after SA. Significant *p*-values (*p* < 0.05).

**Table 3:** The significant differences in means of temporal cortex thickness between the patients after first suicide attempt, repeated.

Hemisphere	Temporal Cortex Part	Mean Cortical Thickness (95% CI) in mm							ANCOVA Age Adjusted,	
		HC (*n* = 54)	First SA (*n* = 29)	Post-Hoc	% Diff.	SA > 1 (*n* = 27)	Post-Hoc	% Diff.		
		1	2	1 vs. 2	1-2	3	1 vs 3	1-3	F_2.106_	*p*
Left	Fusiform	2.78 (2.73–2.83)	2.73 (2.66–2.80)	0.573	1.79	2.67 (2.60–2.74)	0.031	3.90	3.53	0.033
	Inferior temporal	2.93 (2.89–2.98)	2.81 (2.75–2.87)	0.002	4.09	2.78 (2.72–2.83)	<0.001	5.01	11.55	<0.001
	Middle temporal	2.98 (2.93–3.03)	2.86 (2.80–2.93)	0.013	4.02	2.80 (2.73–2.86)	<0.001	6.04	11.29	<0.001
	Superior temporal	2.89 (2.84–2.93)	2.76 (2.70–2.83)	0.006	4.49	2.76 (2.69–2.82)	0.005	4.49	7.61	0.001
	Temporal pole	3.76 (3.67–3.85)	3.59 (3.47–3.72)	0.100	4.52	3.54 (3.41–3.66)	0.015	5.85	4.88	0.009
Right	Superior temporal	2.89 (2.84–2.95)	2.85 (2.77–2.92)	0.995	1.38	2.77 (2.69–2.85)	0.036	4.15	3.27	0.042

HC, healthy controls; SA, suicide attempt. F, *p* values are based on the pairwise comparisons among the means (three groups: HC, first SA, SA > 1). % diff—relative difference describes the difference of the mean cortical thickness between two groups: HC and patients after SA. Significant *p*-values (*p* < 0.05).

**Table 4:** The significant differences in mean volume of hippocampus parts between HC and all study patients.

Hemisphere	Part of Hippocampus	Mean Volume (95% CI) in mm^3^			ANCOVA Age Adjusted	
		HC (*n* = 54)	All Patients after SA (*n* = 56)	% Diff		
		1	2	1-2	F_1.107_	*p*
Left						
	ML	564.42 (545.31–583.55)	536.10 (517.28–554.93)	5.01	2.97	**0.039**
	GC-DG	292.54 (282.38–302.68)	273.57 (263.63–283.52)	6.48	3.79	**0.009**
	CA3	195.78 (188.04–203.51)	183.84 (176.24–191.43)	6.09	4.77	**0.031**
	CA4	250.55 (241.90–259.22)	236.85 (228.38–245.32)	5.46	5.05	**0.027**
	HATA	58.31 (55.76–60.87)	52.94 (50.44–55.44)	9.20	4.46	**0.004**
Right						
	Subiculum	428.21 (411.15–445.27)	404..3 (387.15–420.91)	5.58	3.35	**0.049**
	CA1	640.27 (616.07–664.45)	605.72 (581.90–629.54)	5.39	4.06	**0.047**
	ML	574.35 (553.83–594.88)	545.01 (524.75–565.26)	5.10	3.09	**0.047**
	GC-DG	303.14 (292.17–314.10)	287.19 (276.44–297.94)	5.26	4.25	**0.042**
	HATA	61.01 (58.18–63.84)	56.54 (53.76–59.31)	7.32	5.02	**0.027**

HC, healthy controls; SA, suicide attempt; ML, molecular layer; GC-DG, granule cell layer of dentate gyrus; CA, cornu amonis; HATA, hippocampus–amygdala transition area. F, *p*–values are based on the pairwise comparisons among the means (two groups: HC and all patients after SA). % diff—relative difference describes the difference of the mean hippocampus volume between two study groups: HC and patients after SA. Significant *p*-values (*p* < 0.05) are indicated in bold.

**Table 5:** The significant differences in hippocampus part mean volume between the patients after first suicide attempt, repeated suicide attempts and study controls (first SA, SA > 1 and HC).

Hemisphere	Part of Hippocampus	Mean Volume (95% CI) in mm^3^							ANOVA Age Adjusted	
		HC (*n* = 54)	First SA (*n* = 29)	Post-Hoc	% Diff.	SA > 1 (*n* = 27)	Post-Hoc	% Diff.		
		1	2	1 vs. 2	1-2	3	1 vs. 3	1-3	F_2.106_	*p*
Left										
	GC-DG	292.54 (282.38–302.68)	277.34 (263.49–291.19)	0.247	5.19	269.52 (255.17–283.88)	**0.032**	7.86	3.79	**0.026**
	HATA	58.31 (55.76–60.87)	53.32 (49.83–56.81)	0.072	8.56	52.54 (48.92–56.15)	**0.033**	9.89	4.46	**0.014**
Right										
	Subiculum	428.21 (411.15–445.27)	417.39 (394.12–440.67)	1.000	2.52	389.67 (365.54–413.80)	**0.043**	9.00	3.35	**0.039**

HC, healthy controls; SA, suicide attempt; GC-DG, granule cell layer of dentate gyrus; HATA, hippocampus–amygdala-transition-area. F, *p*–values are based on the pairwise comparisons among the means (three groups: HC, first SA, SA > 1). % diff—relative difference describes the difference of the mean hippocampus volume between two groups. Significant *p*-values (*p* < 0.05) are indicated in bold.

## Data Availability

The data presented in this study are available on request from the corresponding author. The data are not publicly available due to ethical restrictions.
